# Preclinical Assessment of Cardiac Valve Substitutes: Current Status and Considerations for Engineered Tissue Heart Valves

**DOI:** 10.3389/fcvm.2019.00072

**Published:** 2019-06-07

**Authors:** Benjamin L. Zhang, Richard W. Bianco, Frederick J. Schoen

**Affiliations:** ^1^Department of Surgery, University of Minnesota, Minneapolis, MN, United States; ^2^Department of Pathology, Brigham and Women's Hospital and Harvard Medical School, Boston, MA, United States

**Keywords:** heart valve substitutes, tissue engineered heart valves, regulatory pathway, translation, preclinical studies, animal models

## Abstract

Tissue engineered heart valve (TEHV) technology may overcome deficiencies of existing available heart valve substitutes. The pathway by which TEHVs will undergo development and regulatory approval has several challenges. In this communication, we review: (1) the regulatory framework for regulation of medical devices in general and substitute heart valves in particular; (2) the special challenges of preclinical testing using animal models for TEHV, emphasizing the International Standards Organization (ISO) guidelines in document 5840; and (3) considerations that suggest a translational roadmap to move TEHV forward from pre-clinical to clinical studies and clinical implementation.

## Introduction

Implantation of a functional valve substitute (via open surgery or a transcatheter approach) generally improves survival and enhances quality of life of appropriately selected patients with severely diseased heart valves (1, 2). Nevertheless, problems associated with the available devices remain a major impediment to successful long-term clinical outcomes in many recipients. While the design criteria for the ideal replacement valve were outlined initially in the 1950s and 1960s, currently there is no ideal replacement valve that is appropriate for every patient (3). An ideal valve should allow unimpeded forward flow while open, and no regurgitation when closed. Blood flow through the valve should not be turbulent, and it should generate only limited hemolysis and stimulation of the clotting cascade. The valve should be constructed from biocompatible materials with thromboresistant blood-contacting surfaces, and the risk of infection should be low. The valve should also be durable, performing throughout the lifetime of the patient and should not alter her/his daily activities; this means anticoagulation should not be necessary. Moreover, although not mentioned within early objective statements of desirable requirements, for pediatric patients with growth potential, it would be beneficial should the valve be able to enlarge as the recipient grows.

Traditionally and for clinical practice in broad classifications, two types of surgical valves exist: mechanical valves (fabricated from various combinations of polymer, carbon, and metal) and tissue valves (composed, at least in part, of animal or human tissue). Mechanical valves have been used since the first Starr-Edwards valve was introduced in the early 1960's. Contemporary mechanical valves are generally durable but patients with them must receive life-long anticoagulation (with its associated risks of hemorrhage) to mitigate a tendency toward thrombotic complications. On the other hand, surgical tissue valves (almost exclusively today bovine pericardial bioprosthetic valves) do not require anticoagulation in most patients, but instead have a limited durability owing to structural dysfunction mediated by collagen degeneration and calcification of the leaflets. Transcatheter aortic valve implantation (TAVI) has emerged as an attractive therapeutic option for patients with symptomatic severe aortic stenosis who are ineligible or at excessive risk for conventional surgical aortic valve replacement (4).The leaflets of most widely used valves inserted through a transcatheter route are also fabricated from bovine pericardium. Their long-term durability is yet uncertain.

Tissue engineered heart valve (TEHV) technology has been proposed as a potential solution to address the shortcomings of existing mechanical and bioprosthetic valves (5–8). A successful TEHV would comprise a living heart valve replacement, structurally, and functionally analogous to the native valve, without the complications of existing valve substitutes, and with the capacity to remodel, repair, and potentially grow with the patient. The approaches being contemplated to generate a living, functional, and durable heart valve structure can be categorized for simplicity into three distinct paradigms with variants and some overlapping features: (1) seeding cells *in-vitro* on a biodegradable polymeric scaffold and maturing a tissue in a bioreactor to produce a construct consisting usually of a tissue/polymer composite, which is then implanted *in-vivo* (9–12). (2) endogenous tissue restoration, in which a biodegradable porous polymer is implanted without cells or biological adjuncts, inducing *in-vivo* polymer absorption and partial to complete replacement by tissue formation and remodeling, mediated entirely by endogenous cells (13–15), and (3) direct implantation of decellularized valvular or other tissue material. Thus, all paradigms of tissue engineering comprise some combination of a scaffold and cells (seeded *in-vitro* and/or recipient-derived/endogenous cells *in-vivo*); thus, healthy cells are required to create an extracellular matrix (ECM). Some approaches (variants of approach 1 and approach 3) use decellularization to remove cells of either the natural starting materials or the tissue formed *in-vitro* in order to avoid deleterious leaflet changes such as immunological rejection and/or calcification, and promote recellularization with cells entirely from the recipient, intended to assume properties similar to those located in normal valve leaflets. In all cases, following implantation, tissue growth, and remodeling are intended to occur *in-vivo*. Key pathophysiological processes that occur during the *in-vitro* phase (in the formation of a construct in approach 1) and *in-vivo* phases (of all approaches, include cell proliferation and migration, extracellular matrix (ECM) production and organization, scaffold degradation, and tissue remodeling).

## What is the History and Evolution of Medical Device Regulation (With Particular Focus on Heart Valve Substitutes)?

The evolution of medical products regulation in the United States had its origins in the Pure Food and Drug Act of 1906, which was intended to curb interstate markets for adulterated and mishandled food and pharmaceuticals. This legislation required that drugs meet standards of strength and purity, and paved the way for the development of the modern Food and Drug Administration (FDA) in the United States. Nevertheless, fraudulent devices subsequently continued to boast a large range of unproven medical benefits. In 1938, the Federal Food, Drug, and Cosmetic Act of 1938 initially gave the FDA the authority (and responsibility) to regulate medical devices; however, it required only that devices were not misbranded or adulterated. Indeed, there was no system in place for pre-market testing, review, or approval until the 1970s, when the FDA turned its attention to verifying the safety and efficacy prior to marketing of medical devices. The result was the 1976 Medical Device Amendments, (16, 17) considered to be the landmark legislation that initiated the modern era of medical device regulation, which required registration with the FDA of device manufacturers and classified all devices into three classes according to risk. Importantly, this risk-based classification scheme defined the level of approval process needed for marketing of a particular product. Cardiac replacement valves are designated as class III (i.e., devices which “support or sustain human life, are of substantial importance in preventing impairment of human health, or present a potential, unreasonable risk of illness or injury”). Therefore, such devices require pre-market approval (PMA), the most rigorous process required for devices by the FDA, where there is no approved predicate (i.e., existing equivalent) device. In a PMA application, a device must be shown by valid scientific evidence that it is safe and effective in its intended use. Further regulatory requirements accrued with the Safe Medical Devices Act of 1990, which improved post-market surveillance of medical devices, and the FDA Modernization Act of 1997, which created new provisions for pre-market review and permitted use of data from studies of earlier versions of a device in pre-market submission for new versions of the device. Later legislation refined the review processes and regulated advertising for unapproved uses of a product. In parallel, introduction of Good Laboratory Practice (GLP) concepts in the US in 1978 ensured quality with standardized rules in pre-market evaluation. To determine safety and effectiveness of a class III device, the FDA considers the intended use of the device for specific indications, the population for which the device is intended, device reliability, and the risk of device use compared with the likely benefit of device use. In practice, the regulatory framework can be considered in 3 distinct phases of product study: pre-clinical studies, clinical studies, and post-market monitoring. Clinical testing typically consists of a series of studies from first in human use, to large, multicenter prospective, sometimes randomized controlled, “pivotal” trials, of a complexity (number of patients, follow-up time, etc.) determined by the nature of the device and its proposed use. Additionally, post-market procedures require that hospitals and health professionals (and other providers) and manufacturers seek out and report all serious and unexpected adverse reactions to the FDA.

In contrast, the medical device regulatory system in Europe was established in the 1990s and was developed through the European market unification (18). The review system was driven to encourage innovation and to strengthen the European industry. Private, for-profit organizations known as notified bodies regulate medical devices review labeling and testing within the framework of the Medical Device Directives and issues the Conformité Européene (CE) mark, allowing for marketing in EU. The European Commission certifies these notified bodies but in contrast to US regulations, there is no direct oversight by any government body in the premarket review process. Post-market surveillance, however, is conducted by competent authorities (CAs) of each of the EU member states. These CAs are responsible for adverse event reporting, vigilance reporting, and post-market clinical follow-up. Additionally, the European Databank on Medical Devices (EUDAMED) keeps data such as manufacturer registration and certificates as well as exchanges legal information (19, 20). Additionally, although medical device approval in the U.S. requires demonstration of safety and efficacy, medical devices studies in Europe need only demonstrate safety and performance (i.e., perform as designed and benefits outweigh risk). Demonstration of clinical efficacy is not required. Thus, European practices are often considered more “manufacturer friendly” than those in the US.

Pre-clinical studies comprise *in-vitro* (i.e., engineering and materials characterization) and *in-vivo* components (i.e., animal models). In modern practice in the US, animal model testing of heart valve substitutes is guided by document ISO-5840 from the International Standards Organization (ISO), which identifies the goals of pre-clinical *in-vivo* assessment in evaluation of performance characteristics not amenable to be assessed by *in-vitro* testing. Parameters to be studied and their respective protocols as suggested by ISO-5840 are summarized in Table 1, as modified from a previous review (21). With respect to heart valve testing, a critical concept is the requirement of pre-clinical “site-specific” testing of the valve in all the anatomic positions for which the valve is designed, as outlined in the ISO-5840-2 document (22).

**Table 1:** ISO-5840 pre-clinical (*in-vivo*) evaluation guide summary, as modified for tissue engineered heart valves.

**Parameter**	**Protocol**	**Challenges in TEHV**
Species	Same species and gender, similar age and size. Species not specified. Minimum *N* = 10 animals	Unchanged
Site of implantation	Anatomical location as intended for clinical use, i.e., site-specific	Unchanged
Test valve	Clinical quality: relevant size, design, manufacturing, sterilization, and packaging	Unchanged
Control Valve	Passed ISO-5840 standards In clinical use at time of study with >10,000 patients Minimum *N* = 2 animals	No pre-existing control valves Bio-prosthetic valves can be used
Duration	20 week survival for animals Appropriate to study purpose Specified prior to implantation	TEHV will require longer survival times
Technique	Documented reproducible Performed by trained personnel (GLP preferred)	Unchanged
Testing	Hemodynamic profile (echocardiogram, angiography) Ease of handling Audible sound evaluation	Immune-response needs consideration
Serial blood sampling	Preoperative with CBC, electrolytes, coagulation 1 week postoperative Regular interval sampling At euthanasia	Consideration for biomarkers for tissue healing
Pathologic examination	Macroscopic Animal (thromboembolism, pannus formation, inflammatory reaction) Device (structural degradation, calcification) Histological (thromboembolism, degenerative processes, inflammatory reaction)	Requires augmented explanted device examination and histological evaluation (with consideration of detailed cell-matrix-biomaterial interactions, bioresorption, and dynamic mechanical properties)
Test report	Identify animal and valve Preoperative animal condition Detailed surgical and post-surgical course documentation Justification of any deviations from protocol Identity of investigators and institution	Unchanged

Pre-approval/post-approval requirements for device safety and effectiveness information must weigh the appropriate level of testing to permit marketing against potential delays in the availability of potentially life-saving or life-enhancing products for patients. For example, initiation of a clinical study requires an adequate determination of safety based on pre-clinical laboratory, engineering, and animal testing. However, when that is not possible owing to the limitations of pre-clinical test modalities and their ability to predict clinical outcomes, risk, and benefit evaluation must continue in the clinical testing and post-approval settings.

## To What Extent Can Preclinical Testing Ensure the Safety of Tissue Engineered Heart Valves?

Animal testing of cardiovascular devices permits the testing of a heart valve or other device *in vivo* in as clinically relevant situation as possible, thereby providing invaluable information on safety. Nevertheless, these data may not accurately predict device performance and clinical outcomes in pre-market clinical trials and widespread post-market (“real world”) use and over many years' post-implantation. Uncertainty arises because (1) animals used in pre-clinical testing (i.e., animal models) frequently have different anatomy and physiology than humans; (2) with animal models, heart valves or other devices are placed into a normal rather than a pathologic context; and (3) animal thrombotic tendencies and healing kinetics and mechanisms may differ from those in humans. Furthermore, owing to limited subject numbers, subject variability, and post-operative intervals, (4) pre-clinical studies (and even pre-market clinical trials) performed for device approval may not be adequate to detect rare, yet catastrophic adverse events; (5) durability limitations that become significant only after extended implantation over intervals that are longer than those in which animal studies are done; and (6) complications occur post-market that are related to potential performance-affecting factors that can vary among patients (e.g., the effects of extreme mechanical challenges to valve performance such as exercise, or biological effects such as atrial fibrillation or genetic abnormalities of thrombosis or healing). Such low-occurrence events might only be detected in large-sample study populations and over longer follow-up periods, making it necessary to gather detailed information after a product is available for clinical studies (although limited patient numbers are of necessity followed) and in general clinical use. These factors require and justify long and detailed recipient follow-up. Key historic examples of the problems encountered in cohorts of heart valves that were identified and understood only after marketing include the Braunwald-Cutter Cloth-Covered heart valve (23), the Bjork-Shiley 60–70° Convexo-Concave heart valve (24, 25), bioprosthetic heart valve calcification (26), the Medtronic Parallel heart valve (27), the Carbomedics Photo-Fix pericardial heart valve (28), and the St. Jude Silzone valve (29, 30). In each case, clinico-pathologic correlations established by careful analysis of clinical data coupled with implant retrieval and pathological specimen evaluation studies were critical in identifying, understanding, and managing the issue.

Standardized animal models for pre-clinical testing of heart valve substitutes have been reported in dogs, primates, pigs, calves, and sheep. Each model has advantages and limitations, and none of these animal models faithfully and reliably replicate human anatomical and physiological conditions. This is reflected in ISO-5840, which acknowledges that no universally accepted animal model yet exists. For each study, the animal model used should be justified with respect to the characteristics of the model, the anticipated vulnerabilities of design and materials, and potential clinical indications for use.

The use of dog models for valve implantation is largely only of historical interest. In addition to government regulations and social concerns, increased incidence of infections and thrombotic complications has caused this model to fall out of favor (31–33). Primate models most closely resemble humans anatomically and physiologically with low somatic growth, making them favorable for long-term studies. However, these models are much more expensive to purchase and maintain compared to other animals, and they carry important ethical restrictions (34). Instead, in current studies, sheep models are used predominantly, with reports accounting for 78% of the pre-clinical testing of all the models currently used; this has included juvenile, adolescent, and adult sheep (35). The benefits of using sheep models include (though not completely) cardiovascular anatomical structures and physiological functions that are largely similar to those of humans, with similar annulus sizes, equivalent heart rate and cardiac output, moderate somatic growth, availability, and ease of husbandry. There have been controversies regarding thrombotic complications in sheep, but studies generally show no consistent differences between human and sheep coagulation parameters (36–38).

Owing to those controversies for sheep, the porcine model has been utilized in some studies since the platelet activity is close to that of humans. However, porcine models have limitations due to higher infection rates and fast somatic growth of the animal as well as difficulty in maintaining anticoagulation control. potentially leading to hemorrhagic complications (39–42). The growing calf model shares similar limitations due to its fast somatic growth and associated large elevation in cardiac output over the duration of the test period. Nevertheless, because calcification, the most frequent affecting patient outcomes, complication of bioprosthetic valves is magnified clinically in younger patients, calcification mitigation therapies have generally been studied in young, rapidly growing sheep (43).

Animal model studies for two avenues of disruptive heart valve development are particularly difficult: transcatheter valve implantation and TEHVs (44). Difficulty in valve development for TAVI primarily lies in issues with positioning and deployment issues specific to the animal model, and related to the small left ventricular outflow anatomy of sheep and pigs which make it difficult to implant and therefore chronically evaluate transcatheter devices. The native valve in the animal model is also not diseased and thus does not provide the same anchoring conditions for implantation of transcatheter valves as in clinical practice. Additionally, studies using swine model have reported increased elasticity in the annulus, potentiating retrograde migration of a transcatheter aortic valve (45), and para-valvular leak (46). Model development, specifically the placement of an external nitinol band on the ascending aorta, or the creation of a scarred aortic annulus have mitigated this complication and has assisted with experimental implantation to a significant extent. Nevertheless, additional model development work is needed before these modified animal models can be considered standardized.

The remainder of this communication will focus on the specific difficulties associated with pre-clinical studies and clinical translation of engineered tissue heart valves.

## Can the Challenges in Translating TEHV From Pre-clinical to Clinical Studies be Surmounted?

The potential of TEHV is exciting; however, many unanswered questions and substantial challenges of preclinical risk assessment remain before first-in-human (FIH) studies of this technology can be justified (47–49). Although TEHV must be tested in relevant biological systems as the field moves toward clinical application, the use of animal models in experimental valve implantation predated thoughts about TEHV development. The current framework of the ISO-5840 document was developed with only mechanical and bioprosthetic valves in mind. Therefore, the question arises as to whether current standards and regulations are rigorous enough as written to determine pre-clinical safety and efficacy of TEHV prior to clinical trials. With the emphasis is a risk analysis as the core guiding principle of the ISO-5840, the standard is already sufficiently rigorous to guide preclinical protocols, including novel devices and materials such as tissue-engineered valves. Risk analysis determines study design by duration, animal population, and anatomical physiologic considerations for tissue engineered valves with little or no clinical history. Risk analysis also identifies hypothetical hazards from product testing, complaints, adverse events, recalls, audit observations, and other product or process deviations that are involved with the use, misuse, or abuse of the product and is then the summation of each estimate of the risk.

TEHV development triggers the need for extensive animal-based studies with expanded study numbers and extended duration of survival. New mechanisms that drive safety and efficacy considerations may intervene. As all preclinical studies should contain concurrent clinical controls of similar design and construction that are approved and therefore have a clinical history, so too should studies of TEHV have appropriate controls. Thus, we anticipate that approved bioprosthetic valves should serve as these controls in the absence of prior clinical history of TEHV. In addition, the non-regulatory phase of pre-clinical studies will require numerous iterative studies, redesigns, and data analysis between bench and animal phases (and potentially more than those required for conventional mechanical and bioprosthetic valves).

In the progression of clinical testing, it should not necessarily be expected that the final phase of design prior to regulatory phase study (GLP study) will yield 100% uncomplicated devices to achieve preclinical safety. In the authors' experience, there are virtually no bioprosthetic devices that have been complication-free during preclinical testing. At least one of the devices tested will usually demonstrate some biological complication, e.g., thrombus formation or a structural defect. Whether these findings have potential clinical relevance is a judgement determined by pathology analysis of the entire series and comparison with the control group.

For preclinical testing of TEHV, it is essential that only laboratories with relevant expertise with large animal surgery and implantation of a broad range of heart valve types be involved. Regulatory expertise is also mandatory. In this manner, all procedures and personnel, including the surgeon, become control variables. Minimizing extraneous factors allows focus on the experimental device (and the reciprocal responses of the animal) as the sole independent variables. Analysis of explanted devices must be performed by a well-qualified pathological team who has broad relevant experience with the assessment of explanted heart valve substitutes in both humans and in the animal model utilized (50). A non-GLP phase data and conclusions should be submitted to the sponsor with recommendations relative to redesign. Following redesign, the device must undergo additional animal implantation and reiteration to verify the efficacy of condition before proceeding to human studies. Mechanisms of failure must be understood and deficiencies connected.

A successful tissue engineered valve will be dynamic, ultimately composed of specialized viable cells and extracellular matrix (ECM) that can remodel the structure in response to changes in local mechanical forces, and maintain favorable strength, flexibility, and durability, beginning at the instant of implantation and continuing indefinitely thereafter. The formation of engineered tissue depends on inflammation, cell migration, and proliferation, evolution of cell phenotypes, extracellular matrix production, polymer or biological material degradation, and tissue remodeling. An agenda for translating the notion of TEHVs from an extraordinarily interesting experimental methodology to an adopted clinically-useful surgical/interventional tool requires a multifactorial approach. Key areas of uncertainty (and thereby suitable and urgent targets for research and development) include (1) characterization and quality assurance of substrates and tissue constructs, (2) defining animal models, (3) understanding comparative mechanistic and kinetic differences of tissue remodeling between animal model species and humans, (4) developing assays/tools to monitor surrogate and true endpoints before and during function, (5) accommodating potential patient-to-patient and location to location heterogeneity of tissue remodeling processes within the implant, and (6) developing measurable and well-validated biomarkers and surrogates that predict early and extended outcomes.

The challenges that must be surmounted before a TEHV becomes a clinical reality include control of the balance between scaffold absorption (with resultant loss in strength) and new tissue formation (providing strength), which must be predictable and controlled. Clearly there are also other factors and considerations that need to be addressed that are specific to this novel approach and the existing generic issues with conventional heart valve substitutes. Certainly, typical biomaterial-tissue interactions in cardiovascular medical devices, such as thrombosis, infection, and inflammatory interactions, must be acceptable. Another important consideration is whether calcification, the major pathologic process in clinical bioprosthetic valve degeneration, will be problematic in TEHV (26). Thus, although calcification is frequently encountered in experimental heart valves composed of tissue or polymer, especially in the widely-used sheep models, it is uncertain whether this process will be limiting in humans implanted with engineered tissue heart valves. (12, 51, 52).

Animal models for TEHV such as sheep have yielded some promising results. However, further detailed studies will be needed in these models, other animal models, and in human studies. Whether, to what extent, and which animal models are suitable for testing tissue-engineered valves have not yet been determined. Experience suggests that sheep tend to heal and produce fibrous tissue more rapidly and completely than humans; thus, there is controversy over to what extent results from sheep models, widely used in heart valve development, can predict human outcomes. Owing to immunologic considerations, the choice of an animal model for preclinical testing for allogenic or xenogeneic cell-based therapies presents unique challenges. Another key need is the development of science-based approaches to the characterization of TEHV, i.e., measurement of absolute and relative mechanical properties of the scaffold and the tissue-scaffold complex (as they evolve following implantation), characterization of the highly dynamic cell phenotypes and ECM components, and the evolution of the final manufactured product, including shelf-life, stability, and potentially shipping considerations.

A key consideration is that, owing to iterative improvements and an abundance of longitudinal clinical data over several decades, currently available heart valve replacements have predictable behavior in defined recipient cohorts. In contrast, *in-vivo* remodeling and thus performance of TEHV will likely display considerable (and potentially not easily predictable) heterogeneity among patients, owing to four key sources of variability in response to TEHV: (1) effects of genetic variation that qualitatively or quantitatively affect healing mechanisms (presumably non-modifiable), and (2) effects of co-morbidities and age-related changes, including diabetes, chronic kidney disease, and other concurrent conditions, and (3) medications and environmental factors (e.g., diet, smoking), and (4) potential patient variability in the kinetics and completeness of scaffold degradation.

Thus, some patients might not appropriately remodel their tissue-engineered valves, and failure could occur, either by insufficient or insufficiently rapid tissue remodeling (i.e., whereby the composite strength of tissue and substrate could become inadequate to resist the local mechanical stresses) or by an exuberant remodeling (i.e., causing tissue overgrowth, distortion, or stiffening). Thus, to accommodate uncertainties of engineered tissue valves, the usual modalities for demonstrating pre-clinical and clinical safety may need to be altered due to unpredictability of the interactions of the engineered tissue with the recipient's native tissue. Indeed, it may become important to apply principles analogous to those of pharmacogenetics-pharmacokinetics, a field which seeks to understand the mechanisms of individually determined variation in drug metabolism and, to understanding (and potentially controlling) the range of variability (53). Moreover, for approaches that utilize pre-seeding of cells, it will be crucial to understand not only the cellular phenotypes present before implantation, but also whether the seeded cells remain viable and attached to the scaffold following *in-vivo* implantation and how they interact with cells of the recipient.

Additionally, with the current ISO-5840 guidelines in pre-clinical testing, durability testing of TEHV products is a difficult consideration, particularly with regard to degradation. With pre-clinical testing requiring valves to last 200 million cycles in the ISO-5840 guidelines, this may not have the same comparative outcomes due to patient variability as well as the innate durability challenges of TEHVs (i.e., owing to complex tissue-polymer composite structures and dynamic structure during fabrication and *in-vivo* function). While many studies have shown favorable short term mechanical durability *in vitro* and in the animal model, long-term durability has been a challenge in part due to progressive leaflet shortening due to the nature of the contractile cells and other pathologies in the implanted tissue (54). While TEHVs produced using processed xenografts or engineered tissue may be able to demonstrate durability under these requirements, the same expectations may not be able to be met with polymer constructed valves where the design relies on polymer degradation and replacement by tissue over time.

The identification and methods to assay of biomarkers that predict TEHV outcomes post-operatively would facilitate the ability to understand, monitor, and potentially control patient-to-patient differences in tissue remodeling capability *in-vivo* and to monitor evolution of implant structural remodeling and function in an individual to predict implant failure. Thus, conventional and innovative invasive and/or non-invasive anatomic and functional imaging modalities may be important tools to recognize surrogate endpoints for success and failure. Specific molecular biomarkers may be identified and validated by assessing tissue healing and remodeling during *in-vitro* and *in-vivo* experiments; (55) suitable biomarkers will need to be followed *in-vivo*, possibly via chemical assays in the serum or urine or via molecular imaging. Key targets for characterizing tissue-engineered constructs include assays of tissue composition, cellular gene expression and phenotype, whether and what types of stem cells are present, ECM, key effectors of tissue remodeling and tissue quality. These biomarkers should reflect the mechanism of a significant clinical event or long-term outcome. Such an understanding, and the potential ability to measure key biomarkers of outcome – ideally, prior to implantation – could also contribute to more informed patient selection. Moreover, demonstration of long-term safety and efficacy (depending on biocompatibility, durability, modes of failure, and ease of monitoring) of these valves in humans will be a challenge. Thus, risk/benefit relationships of engineered tissue may be less predictable than those of accepted technology.

The potentially unpredictable nature of TEHVs is exemplified by the MatrixP decellularized xenograft valve (56). Despite adequate performance of the product in the animal model, results in clinical use were not favorable. Short and long term results demonstrated failure at the distal anastomosis by intimal proliferation and peripheral narrowing, a phenomenon at which was hypothesized to be a result of the immunological response to the xenograft wall proteins (57). Accordingly, some efforts in TEHV have focused on monitoring of the immune response in the primate model for development of TEHV (58). This aspect of translation from pre-clinical to clinical use is not greatly emphasized in current bioprosthetic valve products and is not considered in the ISO 5840 and will need to be carefully considered with TEHVs. Indeed, since laboratory engineered tissue and decellularized native tissue can have immunoreactive components adverse effects owing to immunological reactions should be considered in the development and use of TEHV. These may arise in the possible mismatch of species form tissue implanted to animal model species or ultimately to the human. An important corollary is that the animal model species used in pre-clinical testing may not precisely predict the response of that product in humans. Additionally, specific cell responses may be critical for TEHV success. For example, macrophage transitions from M1 to M2 phenotypes may be important for healthy tissue regeneration of near-normal architectures rather than fibrous tissue formation (59). This and other considerations in which TEHV may provide novel and poorly understood pathophysiology mandate that, wherever possible, mechanisms involving cells, and matrix biology must be understood, possibly using hypothesis-driven small animal model studies (60).

**Figure 1:**
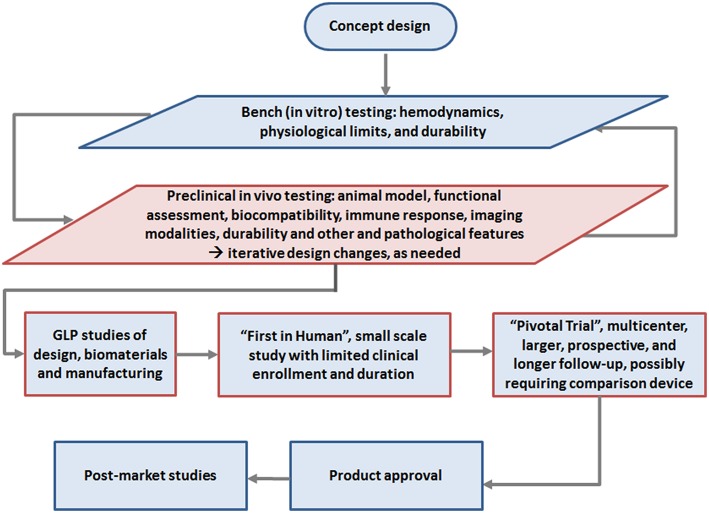
Roadmap of development to clinical use of TEHV, highlighting the pre-clinical study phase emphasized in this review (red box): Initial concept design followed by *in vitro* testing and *in vivo* animal studies, with potentially iterative preclinical testing and redesign steps as indicated (e.g., materials, design, animal model, etc.). Once a final design is reached, *in vivo* animal testing is conducted in the high quality Good Laboratory Practice (GLP) phase for submission to regulatory authorities, i.e., the FDA or European Notified Body for approval for clinical studies. Following this limited “first in human” study, “pivotal” clinical studies should commence with careful long term followup. If pivotal studies demonstrate satisfactory safety, performance and potential efficacy, the end product typically gains approval for marketing.

Ultimately, it is likely that early transition of TEHV technology from pre-clinical to FIH studies will follow guidance for Investigational Device Exemption (IDE) applications for early feasibility studies (61) of significant risk devices, justified by an appropriate benefit-risk analysis and adequate human subject protection measures, in a small number of patients, carefully followed for extended periods. Early feasibility studies would allow for early clinical evaluation of devices to provide proof of principle and initial clinical safety data when available nonclinical testing methods cannot provide the information needed to advance the developmental process. The summary of the development pathway is shown in Figure 1 based on contemporary development of several TEHV concepts (12, 13, 49).

In conclusion, the ongoing clinical interest, academic studies of models and mechanisms and results, and corporate investment in TEHV technology seem justified, given the progress to date and the potential for broad and impactful clinical translation and patient benefit. In the heart valve space, there are applications not only for surgical and transcatheter aortic valve replacement but also for mitral, pulmonary, and perhaps tricuspid valve substitution, via both implantation routes, and across both pediatric and adult populations. Although clinical study is expected to provide information for TEHV that cannot practically be obtained through preclinical experiments, the preclinical studies that provide the focus of the discussion in this communication will remain both challenging and of paramount importance. Understanding and controlling polymer resorption, new tissue formation and remodeling, controlling inflammation and immunological responses, ensuring mechanical stability, minimizing degradation mechanisms such as calcification, and ensuring predictable functionality and overall durability will be the leading areas of concern (47–49). Moreover, many of the considerations raised in this review have relevance for other cardiovascular applications, such as small diameter vascular grafts for peripheral and coronary artery replacement, as well as arteriovenous grafts for renal hemodialysis access (62, 63).

## Author Contributions

BZ and RB wrote the first draft of the manuscript and edited later drafts. FS augmented, edited, and finalized the manuscript. All authors approved the final version.

### Conflict of Interest Statement

FS is a paid consultant to Gore, LivaNova, Medtronic and Neograft, and a paid consultant and Scientific Advisory Board member of Xeltis. The remaining authors declare that the research was conducted in the absence of any commercial or financial relationships that could be construed as a potential conflict of interest. The reviewer ZS declared a shared affiliation, with no collaboration, with one of the authors RB to the handling Editor.
